# Editorial: Digital mindfulness in primary care: enhancing health through technology

**DOI:** 10.3389/fmed.2026.1824821

**Published:** 2026-04-01

**Authors:** Hao Chen, Yehui Tu, SiQi Zeng, Chao Liu

**Affiliations:** 1 School of Film Television and Communication, Xiamen University of Technology, Xiamen, China; 2 School of Journalism and Communication, Hua Qiao University, Xiamen, China

**Keywords:** Digital mindfulness, implementation and ethics, Mobile health (mHealth), Primary Care, Virtual reality (VR)

In recent years, the growing burden of mental health conditions has intensified calls for scalable, accessible, and preventive forms of support across health systems. The World Health Organization has repeatedly emphasized that mental disorders remain a major contributor to global ill health, while service coverage continues to lag behind need, particularly in settings with limited specialist resources. At the same time, primary care has become an increasingly important site for early identification, low-intensity intervention, and continuity of mental health support, especially for common conditions such as depression and anxiety that can often be managed within primary care pathways (1, 2).

Against this background, digital mental health approaches have drawn growing scholarly and clinical attention because they offer the possibility of extending support beyond conventional face-to-face delivery. Within this broader landscape, digital mindfulness—including app-based, web-based, video-guided, and immersive mindfulness interventions—has emerged as a promising avenue for promoting emotional regulation, stress reduction, and psychological wellbeing. Recent evidence syntheses suggest that mindfulness apps and online mindfulness-based interventions can produce beneficial effects for outcomes such as depression, anxiety, distress, and wellbeing, although effect sizes vary and study quality, follow-up duration, and comparator conditions remain important limitations (3, 4).

However, the relevance of digital mindfulness to primary care cannot be reduced to questions of efficacy alone. A primary care perspective also requires attention to reach, engagement, usability, equity, and implementation. Research on digital mental health has shown that user engagement is often uneven and shaped by multiple barriers and facilitators, while broader critical scholarship has highlighted concerns related to data governance, social inequality, and the societal implications of digital mental health technologies. These issues are especially salient when digital interventions are introduced into routine care or deployed across culturally and infrastructurally diverse settings, including low- and middle-income countries (5–8).

It is within this scholarly and practical context that the present Research Topic, *Digital mindfulness in primary care: Enhancing health through technology*, should be situated. The 16 articles collected here reflect a field that is moving beyond simple questions of whether digital mindfulness “works” and toward more nuanced questions of how it works, for whom it works, in which delivery formats, and under what ethical and implementation conditions it can contribute meaningfully to real-world health care. Across clinical trials, observational studies, experimental research, mixed-methods development work, bibliometric mapping, and evidence syntheses, these contributions highlight both the promise and the complexity of integrating digital mindfulness into contemporary health contexts.

## The evolution of digital mindfulness in primary care

Digital mindfulness, therefore, is no longer simply a matter of transferring meditation content onto a digital platform. Rather, it represents an expanding intervention space that includes brief and engaging delivery formats, tailored applications for specific populations, multimodal ecosystems, and immersive technologies such as virtual reality. At the same time, the field is becoming more analytically mature, with increasing attention to mechanisms, heterogeneity, user experience, and implementation feasibility—concerns that closely align with the realities of primary care, where interventions must be both scalable and clinically sensible.

## Articles in this Research Topic

To provide a coherent overview, the 16 articles can be understood as clustering into several complementary lines of work: (1) intervention trials and practice-based clinical studies, (2) technology design and platform-based delivery, (3) evidence synthesis and bibliometric mapping, (4) mechanisms and heterogeneity in digital mindfulness effects, and (5) ethical and global implementation considerations, (6) Digital epidemiology and population mental health contexts.

### Intervention trials and practice-based clinical studies

“*A two-armed pragmatic randomized controlled trial comparing the effectiveness of two self-compassion interventions at reducing perceived stress* ” Kalon et al. compares two self-compassion interventions in a pragmatic RCT design. By focusing on perceived stress reduction in a real-world-leaning trial structure, this work speaks directly to the feasibility and comparative effectiveness questions that matter to primary care implementation (Kalon et al.).

“*The effects of short video app–guided loving-kindness meditation on interpersonal mindfulness, empathy, collaboration, affect, and workplace wellbeing among working professionals”* Liu et al. extends digital mindfulness beyond symptom reduction and into interpersonal and organizational outcomes. Using a short-video app as the delivery vehicle, the study highlights how contemporary media formats can support scalable contemplative practice while targeting interpersonal mindfulness, empathy, collaboration, affect, and workplace wellbeing—outcomes highly relevant to occupational health and preventive mental health strategies (Liu et al.).

“*Improving mental health through body-awareness with dynamic interpersonal therapy in patients with persistent somatic symptoms: an explorative cohort study”* Rovers et al. examines a body-awareness approach embedded in dynamic interpersonal therapy among patients with persistent somatic symptoms. Although not framed purely as “mindfulness training,” its emphasis on body-awareness and mental health improvement resonates strongly with the embodied mechanisms that often underpin mindfulness-based care, especially for somatic symptom presentations in primary care (Rovers et al.).

“*Improvements in physical activity and depression symptoms: an observational study of users of a multi-modal digital mental health platform”* Welcome Chamberlain et al. investigates real-world users of a multimodal platform, linking digital engagement with improvements in physical activity and depressive symptoms. This contributes practice-based evidence on how digital mental health ecosystems may influence both mental and behavioral health indicators (Welcome Chamberlain et al.).

### Technology design and platform-based delivery

“*The development of a mindful eating app for adolescents through mixed-methods feedback from adolescents with overweight or obesity”* Serrano-Gonzalez et al. offers a development-oriented contribution grounded in adolescent feedback. By focusing on mindful eating and using mixed-methods design input, the study shows how user-centered approaches can shape intervention acceptability and relevance—an essential step for sustained engagement in digital interventions for youth (Serrano-Gonzalez et al.).

“*Global educational initiative for managing major depressive disorders in primary care: the MDD minds project”* Dowrick et al. describes a global educational initiative designed for major depressive disorder management in primary care. While not limited to mindfulness *per se*, it demonstrates how education, systems thinking, and international collaboration can scaffold primary care mental health capacity—creating the infrastructure into which digital mindfulness tools may be integrated (Dowrick et al.).

### Evidence synthesis and bibliometric mapping

“*Bibliometric analysis of trends, innovations, and the future of CBT-based mobile interventions for depression”* Gao et al. maps a broader ecosystem of CBT-based mobile interventions, providing a macro-level view of research hotspots and future directions. For digital mindfulness researchers, this type of bibliometric lens helps situate mindfulness/compassion tools within a wider digital therapeutics field (Gao et al.).

“*The effectiveness of virtual reality in enhancing mindfulness: a systematic review and meta-analysis”* Xie et al. synthesizes the growing VR-mindfulness evidence base. The review is especially valuable for primary care translation because VR interventions often face questions of cost, accessibility, and workflow fit; a meta-analytic summary helps clarify the strength and boundaries of current evidence (Xie et al.).

“*Virtual reality for the management of musculoskeletal pain: an umbrella review”* Kalikanov et al. focuses on VR for pain management. Given the high prevalence of musculoskeletal pain in primary care and the close coupling between pain and psychological distress, this umbrella review complements mindfulness-focused VR work by detailing evidence trends across an adjacent, clinically central domain (Kalikanov et al.).

“*Effectiveness of mobile health in symptom management of prostate cancer patients: a systematic review and meta-analysis*” Chen, H. S., et al. extends the conversation to cancer symptom management. While broader than mindfulness alone, it strengthens the clinical rationale for leveraging mobile health in chronic illness contexts where stress regulation and coping support are often needed (Chen, H. S., et al.).

### Mechanisms and heterogeneity in digital mindfulness effects

“*How digital mindfulness training promotes meaning in life among social workers: a parallel mediation analysis and latent profile analysis”* Rui et al. explicitly models *how* digital mindfulness may work, combining parallel mediation with latent profile analysis. This dual approach is a methodological strength: it simultaneously tests mechanisms and acknowledges heterogeneity—two elements that are increasingly necessary for translating digital mindfulness into targeted, efficient interventions (Rui et al.).

“*Procrastination and online social anxiety: the mediating role of cognitive overload and the moderating influence of mindfulness”* Chen, S., et al. examines mindfulness as a moderating influence within a digital-behavioral pathway (cognitive overload → social anxiety-related outcomes). It contributes to a growing body of work positioning mindfulness not only as an intervention outcome, but also as a psychological resource shaping how individuals navigate digital demands (Chen S., et al.).

“*The effects of mindfulness on psychological thriving among urban park visitors: evidence from three experimental studies”* Meng et al. broadens the setting to urban nature exposure with experimental evidence. Although not “primary care” in the clinic sense, thriving and positive mental health are increasingly recognized as prevention targets; this work offers insight into how mindfulness can intersect with everyday environments that primary care often recommends (e.g., lifestyle and behavioral prescriptions) (Meng et al.).

“*Effect of digital mindfulness on perceived stress and anxious emotion among college students”* Xiong et al. provides evidence on stress and anxious emotion outcomes in a student sample—an important population for early intervention and prevention. The findings contribute to understanding where digital mindfulness may be most impactful and how outcomes manifest in high-demand life stages (Xiong et al.).

### Ethical and global implementation considerations

“*Bioethical Considerations in Deploying Mobile Mental Health Apps in LMIC Settings: Insights from the MITHRA Pilot Study in Rural India*” Navarro-Aguirre et al. addresses a critical frontier: ethics and governance in LMIC deployment. For primary care, this is not a peripheral issue—data privacy, consent, risk management, digital literacy, and equity can determine whether an app-based solution benefits communities or amplifies vulnerabilities. This contribution anchors the Research Topic in an urgently needed ethical conversation (Navarro-Aguirre et al.).

### Digital epidemiology and population mental health contexts

“*Correlates of antenatal anxiety: smartphone use, depressive symptoms, and hypertensive disorders in a cross-sectional study in Southwest China”* Guo et al. situates smartphone use within maternal mental health and medical correlates. Such evidence is important because primary care frequently encounters anxiety in antenatal populations, and digital behaviors may both signal risk and provide channels for support (Guo et al.).

## The significance of digital mindfulness for primary care

Taken together, the contributions in this Research Topic underscore three broader insights. First, digital mindfulness is diversifying: delivery modalities now range from short-video platforms to VR, and from single-focus apps to multimodal ecosystems. Second, the field is maturing methodologically: researchers are increasingly combining pragmatic trials, real-world observational evidence, experimental designs, and advanced modeling of mechanisms and heterogeneity. Third, implementation and ethics can no longer be treated as afterthoughts—especially when digital mental health tools are deployed across unequal infrastructures and culturally diverse settings.

Ultimately, digital mindfulness in primary care should not be framed merely as a convenient substitute for face-to-face programs. Instead, it can be understood as a flexible, scalable, and potentially personalized layer of preventive and supportive care—one that must be evaluated not only for efficacy, but also for safety, equity, usability, and fit within real clinical workflows.

## Conclusion

As we conclude this Research Topic on “Digital mindfulness in primary care: Enhancing health through technology,” we extend our sincere appreciation to all authors, reviewers, and editors who contributed to this collection. The work presented here reflects a field in rapid development—moving beyond proof-of-concept and toward clinically relevant, ethically grounded, and system-aware approaches to digital mental health support. We hope this Research Topic will stimulate further interdisciplinary collaboration and help advance digital mindfulness as a credible, responsible, and effective pathway for enhancing mental health within and beyond primary care.
